# Limitations and advances in new treatments and future perspectives of corneal blindness

**DOI:** 10.5935/0004-2749.20210042

**Published:** 2021

**Authors:** Rosalia Antunes-Foschini, Leidiane Adriano, Adriana de Andrade Batista Murashima, Amanda Pires Barbosa, Luis Fernando Nominato, Lara Cristina Dias, Marina Zilio Fantucci, Denny Marcos Garcia, Monica Alves, Eduardo Melani Rocha

**Affiliations:** 1 Department of Ophthalmology, Otorhinolaryngology and Head and Neck Surgery, Faculdade de Medicina de Ribeirão, Universidade de São Paulo, Ribeirão Preto, SP, Brazil; 2 Discipline of Ophthalmology and Otorhinolaryngology, Faculdade de Ciências Médicas, Universidade Estadual de Campinas, Campinas, SP, Brazil

**Keywords:** Blindness, Corneal diseases, Corneal transplantation, Genetic therapy, Celland tissue-based therapy, Stem cells, Cegueira, Doenças da córnea, Transplante de córnea, Terapia genética, Terapia baseada em transplante de células e tecidos, Células-tronco

## Abstract

This review is intended to describe the therapeutic approaches for corneal blindness, detailing the steps and elements involved in corneal wound healing. It also presents the limitations of the actual surgical and pharmacological strategies used to restore and maintain corneal transparency in terms of long-term survival and geographic coverage. In addition, we critically review the perspectives of anabolic agents, including vitamin A, hormones, growth factors, and novel promitotic and anti-inflammatory modulators, to assist corneal wound healing. We discuss the studies involving nanotechnology, gene therapy, and tissue reengineering as potential future strategies to work solely or in combination with corneal surgery to prevent or revert corneal blindness.

## Abstract (pt)

O presente trabalho traz uma revisão das abordagens terapêuticas para a cegueira da córnea. O estudo detalha as etapas e os elementos envolvidos na cicatrização da córnea. Ele mostra as limitações das estratégias cirúrgicas e farmacológicas usadas para restaurar e manter a transparência da córnea em termos de sobrevida a longo prazo e alcance geográfico. As perspectivas dos agentes anabólicos, incluindo vitamina A, hormônios, fatores de crescimento e novos moduladores pró-mitóticos e anti-inflamatórios para auxiliar a cicatrização da ferida na córnea, são revisadas criticamente. Aqui, apresentamos estudos envolvendo nanotecnologia, terapia gênica e reengenharia de tecidos como possíveis estratégias futuras para atuar de maneira isolada ou combinada com a cirurgia da córnea para prevenir ou reverter a cegueira corneana.

## INTRODUCTION

In the first part of this review, we challenge the common sense of three assumptions concerning corneal blindness and reinforce that a) corneal blindness is not a minor epidemiologic problem; b) although the major causes are predictable, the current prevention measures against corneal blindness are not followed or not effective; and c) corneal transplantation, which is the major therapeutic strategy, is limited in terms of access and long-term effectiveness, which is because approximately 180,000 corneal transplants are performed per year across the world; however,16 million people are blind due to corneal diseases and the average half-life of a corneal transplant is lower than 15 years^(1-4)^. In the second part, we review alternative therapeutic approaches to corneal transplantation to treat corneal blindness, including the modalities of lamellar keratoplasty, ocular surface reconstruction, and potential novel medications designed to modulate corneal wound healing. For this purpose, we conducted a literature review of recent medical articles. The mechanisms underlying corneal wound healing and therapeutic approaches to prevent or treat corneal blindness were addressed with variable completeness, depending on the uniqueness and relevance, based on an extensive search of the literature. Therefore, in this paper, we intend to offer a review of the state-of-the-art corneal blindness treatment approaches, adding a critical evaluation of the clinical relevance, whenever possible^(5)^. This is justified by the fact that reverting corneal blindness by corneal transplantation is a limited strategy, as mentioned earlier. In summary, this review demonstrates the alternative corneal surgical modalities and their limitations and investigates the perspectives of novel therapeutic strategies for corneal blindness based on the current understanding of corneal wound healing.

### Lessons learned from the past

In the XIX century, a Brazilian ophthalmologist, Gama Lobo, reported about a disease in four children, slave descendants, with infections involving the lungs, mucosal tissues, and eyes. The children were very thin and weak and cried without producing tears. He named the new disease as *Ophthalmia Braziliana* and hypothesized that it was caused by eating few meals or being deprived of essential nutrients in the food^(6)^. In 1934, after the discovery of vitamin A, Mellanby demonstrated that rats deprived of this vitamin for 10 days showed an absence of tears, corneal melting, and, just as importantly, degeneration of trigeminal ganglions (TGs)^(7,8)^. Several items must have coincided for an eventual lethal outcome in the patients described by Gama Lobo, but a key nutritional element required for vision, corneal integrity, and body health was found to be vitamin A or retinoic acid, and the condition associated with its deprivation is known as keratomalacia^(6,8-10)^.

Vitamin A supports not only the corneal tissue but also the lacrimal functional unit (LFU) that protects the cornea^(11)^. Vitamin A deprivation may be a health problem in the XXI century, whereas the application of this nutrient could be an adjuvant topical anabolic therapy for corneal wound healing, indicating two hypotheses that require further investigation^(12,13)^.

In Sweden, the ophthalmologist Henrik Sjögren described a series of 19 female patients with inflammation of the ocular surface as having tear deficiency, dry mouth, and, in some cases, polyarthralgia. He termed this condition as *keratoconjuctivitis sicca,* and decades later, it was redefined as a systemic disease named after him as Sjögren’s syndrome (SS)^(14,15)^. SS is one of the most common autoimmune diseases worldwide^(16,17)^. The etiology of this disease remains unknown, and no possible cures have yet been developed^(18,19)^. However, several studies have clarified that inflammatory events occurring in the ocular surface and in the lacrimal gland (LG) and tear deficiency are associated with hormonal status and the state of the neural network, confirming the model of LFU^(11,20,21)^. Furthermore, in severe cases, SS can induce corneal melting or opacity *per se*^(22-24)^. Since its first description, a clear aspect about SS is its predominance in women and the role of sex hormones in its physiopathology, emphasizing the prospect of the therapeutic use of androgens and other anabolic hormones for ocular surface diseases^(25)^.

The above-described lessons teach us two points; first, the neural network integrates the cornea and the LG by the sensorial and autonomic nerves in the LFU. It maintains the constitutive and regulated exocrine secretion, including anabolic agents such as hormones, vitamin A, and growth factors, which are crucial for corneal integrity and homeostasis. Second, the anabolic agents and growth factors present in the LFU are useful in the therapeutic approaches to prevent or treat corneal blindness.

### Corneal wound healing mechanisms

To understand the role of growth factors and anabolic agents in preventive and therapeutic approaches for corneal diseases, it would be helpful to review the steps and the players involved in the process of corneal wound healing. The cornea is a transparent organ in front of the eye, with a spherical toroidal or aspheric format and an average central thickness of 520 µm and an average peripheral thickness of 650 µm. Although it possesses such a fragile profile, being almost 90% transparent and typically composed of water, it works as a shield for the eye globe^(26)^. The protective role provided by the tear film is broadly recognized and described as deficient in keratomalacia, SS, and children’s dry eye, where tear deficiency is an early manifestation and the outcome is corneal opacity or perforation^(22,27-29)^.

Corneal restoration during wound healing exhibits the following five properties: a) avascularity, b) high sensitivity, c) epithelial renewal supported by limbal stem cells, d) a distinct corneal layer wound response, and e) cross-talk between the cornea and the LFU^(30-32)^.

**Figure 1:**
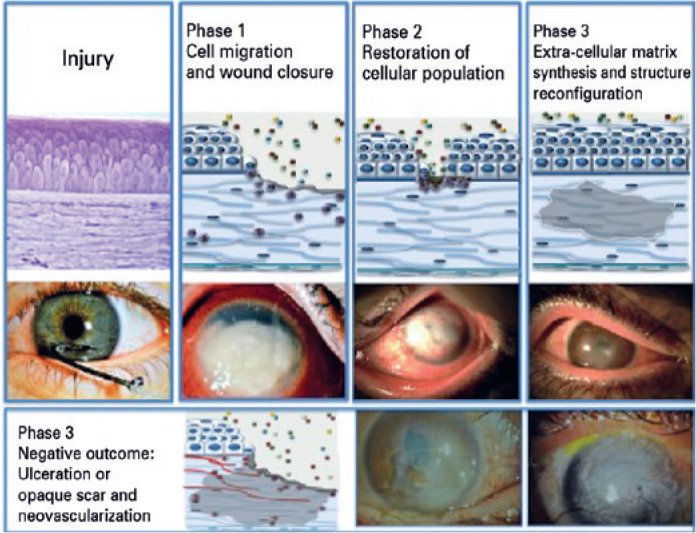
Schematic and clinical illustration of the three phases of the corneal wound healing process.

Corneal wound healing can be divided into three phases^(33,34)^ (Figure 1). In the first step, the hyperacute phase, the cornea loses mass and integrity. The proinflammatory storm is characterized by the secretion of chemotactic factors. Corneal necrosis and clearance occur by collagenolytic destruction and leukocyte permeability and attraction. The symptoms in this phrase include pain and blurred vision. The process is initiated in the first 12 h and may last approximately 7 days, with ocular surface inflammation (redness, tearing, and discomfort) and opacity and the wound being covered by fibrinoid material, building a matrix for the second phase^(33,35-37)^.

The second, subacute phase occurs between an average of 7 to 21 days after the trauma. This phase can be identified using typical biomarkers, viz., keratocyte and epithelial cell proliferation. The inflammatory signs are milder, and anabolic and growth factors and anti-inflammatory cytokines comprise the predominant early mediators of inflammation^(38-40)^.

In this phase, the adjacent healthy epithelial cells lose the structures that make them a compact and interconnected layer (tight junctions and hemidesmosomes) and migrate to cover the wound. These corneal epithelial cells provide paracrine secretion, produced by the epithelial cells or filtered from the tear film that are now regulated to carry anabolic agents and growth factors to induce the extracelluar matrix reconstitution^(38,41)^. This process induces keratocyte mitosis and dedifferentiation in myofibroblasts or fibroblasts, depending on the interactions between cytokines and growth factors^(42)^. Fibroblast growth factor-2 (FGF-2) is associated with cell proliferation in the wounded cornea, and transforming growth Factor-β (TGF-β) is associated with the synthesis of the fibrotic extracellular matrix and keratocyte dedifferentiation, which induces faster and stronger, but also more opaque, corneal scars^(43)^. Insulin-like growth factors I and II (IGF-I/II) and also insulin in pharmacological levels are capable of synthesizing collagen and proteoglycan, combining the elements into a more organized extracellular matrix, resulting in a more transparent stroma^(42,44)^. During this phase, the inflammatory signs and symptoms reduce gradually and the visual symptoms of visual haze and glare persist.

The third phase is initiated by the 3^rd^ week and lasts for several months and is characterized by extracellular matrix tissue remodeling and homeostasis recovery, including transparency, surface regularity, and the shielding function of the cornea, thus consolidating the healing process. This phase includes edema reduction, collagen secretion, and restoration of nerve fibers, basal membrane, intercellular channels, and epithelial cells. It is marked by symptom attenuation and visual acuity improvement^(33,45)^.

**Figure 2:**
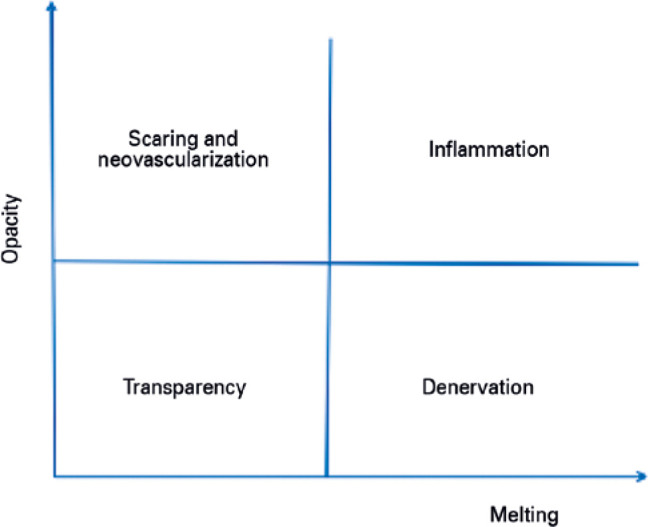
Quadrant representation of the progression of corneal wound healing with one favorable (homeostatic) and two unfavorable outcomes (opaque scar or melting and perforation).

The outcome is dependent on the severity and persistency of the aggression and a combination of external and systemic factors^(33,34,45)^. In the first phase, poor outcomes include progressive stromal erosion, perforation, and corneal melting. In the second and third phases, the process may result in intense and deep opacity, neovascularization, and altered neural network replacement (Figure 2). In these cases, loss of the optic function of the cornea and persistent pain and inflammation are observed in the clinical setting^(33,46)^.

**Figure 3:**
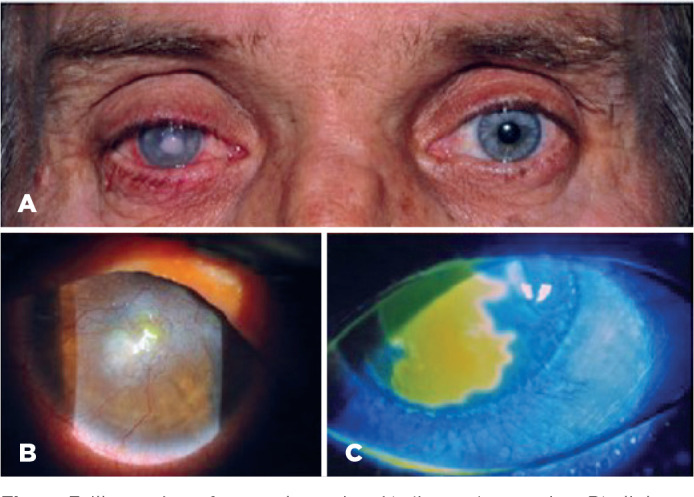
Illustration of corneal opacity: A) direct observation, B) slit lamp image of a corneal scar with neovascularization, and c) the presence of an epithelial defect limited by fluorescein staining. Although none are favorable for vision improvement, both distinct outcomes can occur (i.e., neovascularization and scarring versus chronic epithelium defects and corneal ulceration), although the reasons and mechanisms for their differences are unknown.

These unfavorable outcomes are present in several diseases and also account for the prevalence of corneal opacity and blindness (Figure 3).

### Alternatives to corneal transplantation and novel treatments for corneal opacity and their limitations

In this section, we review the surgical alternatives to fix corneal diseases that cause changes in its shape and transparency. The alternatives range from the less invasive and preventive techniques to the most invasive and applied in severe cases. In the second part, we review the currently available options in topical drug therapy.

#### Surgical alternatives to corneal transplantation

Changes in corneal shape, also known as ectasia, can cause blindness, which does not necessarily result in corneal opacity but induces blindness due to severe refractive problems. Keratoconus is the major type of ectasia whose frequency in the population varies from 0.4 to 86 cases per 100,000 inhabitants^(47)^. The cause of keratoconus is unknown, but it is probably multifactorial. Although keratoconus does not frequently induce corneal opacity or neovascularization, it disturbs the curvature, and biomechanical properties of the cornea, potentially leading to bilateral visual impairment and blindness, making it one of the most frequent reasons for corneal transplantation^(48,49)^. Briefly, conservative treatments include glasses, hard contact lenses, and intrastromal corneal rings, before its severity reaches the need for corneal transplantation. All these treatments are capable of reverting the blindness caused by keratoconus, and more recently, the corneal crosslinking induced by ultraviolet light and riboflavin (vitamin B 2) topical application is being advocated as a strategy to prevent the progression of keratoconus. Despite the high prevalence and the impact of keratoconus on the patient’s life, access to these treatments is hindered by the economic and technological barriers. Furthermore, their long-term efficacy and stability are modest, considering that the disease manifests at a young age and the need for lifetime support^(50,51)^.

#### Alternative techniques to penetrating transplantation

Lamellar corneal transplantations, replacing only the altered layers, constitute a group of growing alternatives to penetrant keratoplasty (PK). These types of transplantations were conceived by Barraquer in Colombia in the 1960s^(52)^. Currently, both the anterior (deep anterior lamellar keratoplasty, DALK) and the posterior modalities, Descemet’s membrane endothelial keratoplasty (DMEK) and its variations, of these lamellar techniques are in use and replacing PK in the majority of referral centers throughout the world^(53-56)^. Clinical trials and meta-analysis conducted till date have demonstrated similar outcomes and prognoses of PK compared to those of lamellar corneal transplantations for treating common corneal diseases, such as pseudophakic bullous keratopathy (PBK), and the same challenges of PK: inflammation and vascularization^(49,55,57,58)^. Studies have also reported promising results for endothelial lamellar corneal transplantations compared with PK in terms of visual acuity, final refractive error, less invasiveness, graft survival, and recovery period^(59,60)^. These modalities replace the corneal endothelium that does not regenerate spontaneously in humans. However, endothelial lamellar corneal transplantations cannot overcome the two major limitations in reverting corneal opacity and blindness at the population level, i.e., the scarcity of corneas for grafting and the dependence on highly specialized centers to provide the treatment^(59,60)^.

#### Ocular surface reconstruction and keratoprosthesis

The pioneering studies of Thoft and Friend conducted during the 1970s opened the possibility of promoting the epithelial regeneration of the cornea^(61)^. The concepts developed from their studies were translated and applied to ocular surface reconstruction for critical cases involving neovascularization, fibrosis, and limbal deficiency^(62-67)^. In addition, the usefulness of the amniotic membrane and *ex vivo* corneal limbal epithelial stem cell expansion was demonstrated in further studies^(68,69)^.

At the same institution where Thoft and Friend started the project for ocular surface reconstruction, but a little early, Doane et al. initiated studies to produce a keratoprosthesis capable of replacing the central cornea, which can be applied in very severe cases of ocular surface scarring with anatomical and functional damage^(70)^.

Both ocular surface reconstruction and its alternative for treating corneal blindness in the most severe cases of surface scarring, the keratoprosthesis, gained technological adjustments and resources in the past few decades. Besides the necessary training and sophisticated equipment, these strategies had limited survival curves in terms of maintaining transparency, visual acuity, or eye globe integrity^(71-75)^. The survival curve of the Boston keratoprosthesis indicated a half-life of 3 years, and that of a limbal transplant from an allogeneic donor was less than 1 year^(71-73,76,77)^. Therefore, ocular surface reconstruction with allogeneic limbal stem cell transplantation and keratoprosthesis for reverting corneal blindness are hampered by limited survival. However, when autologous limbal transplant was possible from grafts obtained from the healthy contralateral eye, the survival was longer than 5 years in more than 80% of the cases^(73)^. Nevertheless, autologous limbal transplant is much less common in clinical practice because of its much less requirement. Transplantation of cultivated limbal epithelial cells and small limbal autologous transplants have been used to avoid the limitations of the scarcity of autologous epithelial stem cells in the damaged cornea or the immune reactions of allogeneic cells; however, again, these techniques are restricted to higher technology centers and selected cases of ocular surface diseases and still require long-term analysis^(78-81)^.

#### Corneal neurotization and other grafting strategies

As indicated previously (Part I), corneal trophism and avascularity are typically sustained by robust corneal innervation^(82-84)^. Loss of innervation leads to fragility in corneal transparency^(83)^. In this context, a surgical technique (neurotization/neurotisation, as it appears with both spellings in the medical literature) has been proposed to restore corneal innervation and revert neurotrophic keratitis^(85)^. Neurotization is a surgical procedure in which autologous nerve tissue grafting between the neurotrophic cornea and the peripheral nervous system is intended to restore corneal sensation^(86-89)^.

Other grafting strategies involve the salivary gland duct transposing to the OS or minor salivary gland grafts to the orbital cavity to provide basal biological fluid to regenerate and sustain the epithelial surface^(90-94)^. However, the confidence in this surgical strategy to revert corneal blindness is limited by the lack of controlled trials and long-term results.

#### Topical drug therapy for corneal blindness

The major pharmacological strategies used to prevent and treat corneal blindness as a single or adjuvant treatment include anti-inflammatory, anabolic and growth factors, and neurotrophic and neurotransmitter analogs.

Topical corticosteroids are hazardous options in cases of corneal infection, severe inflammation, and delayed wound healing^(95-98)^. However, topical corticosteroids are still the best choice to prevent corneal transplant rejection and subsequent failure^(99)^. Corticosteroids modulate inflammatory cytokines, thereby reducing neovascularization and opacity^(95)^. Therefore, excluding the contraindications, corticosteroids remain the gold standard adjuvant therapy for modulating corneal wound healing.

Among the natural biological fluids with anabolic, lubricant, and nutritional properties for treating corneal diseases and promoting wound healing in the most se vere cases are the autologous serum (AS) and platelet-rich plasma (PRP)^(100-104)^. However, due to the lack of similar comparative parameters for analyzing the outcomes of several studies together and the short duration of most of the clinical trials, it is not possible to conclude that any of the abovementioned fluids are superior therapeutic strategies^(105,106)^.

The topical use of recombinant nerve growth factor eye drops to restore the neural network in neurotrophic keratitis has been investigated for several years and was recently approved for commercial use as Oxervate^®^ (Cenegermin)^(107-109)^. The other topical medication is ReGeneraTing Agent (RGTA)^®^, a tissue protector that mimics the extracellular matrix and speeds up the corneal wound healing process in refractory conditions by binding with healing agents and protecting against lytic enzymes^(108)^. Based on the limited and short-term controlled observations, the variability of the surgical techniques and the short 8 weeks of observations of the topical therapies (Cenegermin and RGTA), these approaches are being received with caution, and the reports indicate that further studies are required in terms of neurotrophic keratitis, which, as previously mentioned, is one of the most challenging causes of corneal neovascularization and opacity and where corneal transplantation has a very limited prognosis^(49,109-111)^.

The abovementioned descriptions indicate that corneal blindness, and its various causes, cannot be largely reverted by PK or its surgical alternatives in combination with or replaced by adjuvant drug therapy in terms of large-scale or long-lasting strategies^(1,2,57,112-114)^ (Table 1). The lessons learned from the past as mentioned above (Session 2) indicate that vitamin A deficiency is probably not just a cause of dry eye and corneal melting but also disrupts the neural network, which is a crucial support for corneal integrity and still extremely difficult to restore with the current therapeutic strategies as discussed above. Furthermore, in conditions where the tear film is missing (dry eye), not just dryness but also suppression of the protective mediators present in tears, including growth factors and hormones, results in delay or induces a scarring corneal wound healing.

**Table 1:** Current surgical and clinical alternatives for the treatment of corneal opacity and its limitations.

Category		Treatment	Limitations	Author, year	
Surgical		Penetrant keratoplasty (PK)	Availability of corneas to all cases of corneal blindness; limited survival curve in severe cases and reoperations.	Pascolini, Mariotti, 2012^(115)^; Gain et al., 2016^(1)^; Dandona et al. 1997^(116)^; Coster et al., 2014^(56)^; Tan et al., 2018^(117)^	
		DALK	A healthy host endothelium is needed, similar survival curve as PK.	Reinhart et al., 2011^(118)^; Borderie et al., 2009^(3)^; Keane et al., 2014^(58)^	
		DSAEK/DSEK	Graft detachment and primary graft failure, lower optical quality, and faster endothelial loss compared with PK.	Lee et al., 2009^(119)^; Anshu et al., 2012^(120)^; Nanavaty et al., 2014^(57)^	
		DMEK	Surgical complexity in graft preparation and handling, superior results compared with DSEK. Similar outcome and survival curve as PK.	Anshu et al., 2012^(120)^; Price, Price, 2013^(121)^; Tourtas et al., 2012^(122)^; Navanaty et al., 2014^(57)^; Li, et al., 2017^(123)^	
		DWEK	Longer time for recovery. Lack of comparative studies.	Davies et al., 2017^(124)^; Kymiois et al., 2017^(125)^	
		Ocular surface reconstruction with donated limbal stem cells and amniotic membrane	Donor stem cells for bilateral cases, limited survival curve.	Rama et al., 2010^(73)^; Santos et al., 2005^(71)^; Daya et al., 2005^(126)^	
		Keratoprosthesis	Glaucoma, secondary infection, extrusion. Limited survival curve.	Nguyen, Chopra, 2014^(127)^; Basu et al., 2014^(128)^; Al Arfaj, 2015^(129)^; Aravena et al.,^(130)^	
	Clinical	Corticosteroids in the treatment of bacterial corneal ulcers	Controversial, with no definitive evidence to guide treatment decisions.	Carmichael, et al., 1990^(131)^; Srinivasan, et al., 2009^(132)^; Hindman, et al., 2009^(133)^; Wilhelmus, 2002^(134)^	
		Allogeneic serum eye drops for the treatment of dry eye in patients with chronic graft-versus-host disease	Care should be taken to avoid the risk of blood-borne diseases. Need do adhere to guidelines for obtention, preparation, storage, and usage of hemoderivates.	Na, Kim, 2012^(135)^	
		Nerve Growth Factor Recombinant eye drops	Indicated for neurotrophic keratitis. Expensive and limited experience.	Pflugfelder et al., 2019^(109)^	

DALK= deep anterior lamellar keratoplasty; DSAEK= Descemet’s stripping automated endothelial keratoplasty; DMEK= Descemet’s membrane endothelial keratoplasty; DSEK= Descemet’s stripping; DWEK= Descemetorhexis without endothelial keratoplasty.

### Future perspectives of corneal blindness: drugs, cell genetic reprogramming, tissue reengineering, and combined strategies

After identifying that treatment is not simple or widely accessible and that the cure is not possible in several cases due to the time restrictions of the treatments, it is necessary to identify the pathophysiological events associated with corneal opacity. Destruction of the cornea occurs in one of the following two ways: a) melting and perforation caused due to inflammation and necrosis and/or b) scarring and neovascularization caused due to denervation. Depending on the intensity of each process, it may cause corneal damage to one of the poles (ulceration or neovascularization) or restrict it somewhere between the two (Figure 3). Therefore, inflammation and denervation are the events that need to be reverted to prevent corneal blindness.

The present knowledge about the therapeutic options to assist corneal wound healing to prevent or revert corneal blindness is detailed below in the following topics: a) regenerative drugs (growth factors and hormonal agents); b) novel analgesic and anti-inflammatory drugs delivered as eye drops or using c) nanotechnology; d) cell genetic reprogramming (*e.g*., viral vector gene transfer or other strategies of gene therapy) of the cornea or its natural delivery system, the LG; e) tissue reengineering (*e.g*., combined allogeneic transplantation, including embryonic tissues); and combined approaches.

#### Regenerative drugs

Sex and other hormones are involved in the maintenance of the cornea and the ocular surface and in the response to diseases^(25)^. Estrogens elevate the inflammatory response in the LGs of female individuals compared to that in male individuals of several species^(136)^. In contrast, androgens, insulin, and other hormones exert anti-inflammatory and anabolic effects on the cornea and LGs^(25)^. Diseases involving the absence or impaired action of hormones that risk compromising the transparency and integrity of the cornea include diabetes mellitus (DM) and thyroid autoimmune disease, among others^(25,137)^. Therefore, the therapeutic use of hormones may assist the process of corneal wound healing and restore the ocular surface homeostasis.

The anabolic effects of growth factors, such as NGF and IGF-I, and hormones, such as insulin and androgen topical therapy, include improvement of tear secretion and reduction of the duration of ulcers^(109,138-142)^. Of interest, the healthy LG is not only a target but also has the capacity to produce and secrete growth factors and hormones such as insulin and convert testosterone into a more powerful hormone, dihydrotestosterone, by type 1 and 2 5-alfa-reductase^(143,144)^.

The conceptual support for using insulin as a topical corneal therapy is based on the observation that DM induces neurotrophic keratopathy and causes slower wound healing, lower tear secretion, and changes in the cornea and LG structures^(145-147)^. Insulin deprivation leads to LG malfunction and corneal damage, and it has been observed that topical or systemic insulin replacement can restore tear flow and the corneal structure in diabetic human and animal models^(148-152)^.

As mentioned in section 3, insulin has a corneal wound healing property compared with keratocytes that is not as rapid as that exhibited by growth factors, including IGF1, but is less scarring^(141,153)^. Studies have suggested that insulin could be used as a supportive treat ment to prevent corneal diseases in diabetic subjects and as a potential promoter of corneal wound healing in patients with dry eye disease^(152,153)^.

Studies conducted using diabetic animal models have demonstrated that insulin topical therapy could improve neurotrophic corneal ulcers and dry eye disease; however, a recent clinical trial in humans revealed that insulin topical therapy showed similar outcomes as those of artificial tears after 4 weeks of treatment^(150,154,155)^. The limiting aspects pertaining to the storage and delivery of the small and unstable insulin peptide to the ocular surface have been addressed using nanotechnology, where the number of microparticles, stably enveloping the therapeutic molecule, and the time to modulate the wound healing process can lead to a promising strategy for treating corneal diseases and dry eye disease^(150,156)^.

In the inner face of the cornea, the topical use of Rho kinase inhibitors restored endothelial pump function and reduced edema in PBK when used as a single or adjuvant treatment in combination with various modalities of deep lamellar corneal transplantation^(157,158)^. This topical corneal treatment increased the endothelial cell density and was able to minimize the waiting period for a corneal transplant and replaced it with lower risk procedures^(157)^.

#### Novel analgesic and anti-inflammatory drugs

Recent studies have demonstrated that cannabinoid analogs can reduce pain sensations and leukocyte migration to corneas burned with silver nitrate^(159,160)^. As these outcomes were shown to be comparable or superior to those of topical corticosteroids in reducing corneal pain, inflammation, and opacity without causing the side effects of ocular hypertension and corneal toxicity associated with topical steroids and nonsteroidal anti-inflammatory drugs (NSAIDs), cannabinoid analogs could be considered as a useful adjuvant corneal topical therapy that require further studies^(22)^. Of interest, in 2020, the Brazilian Health Surveillance Agency approved the therapeutic use of cannabidiol for treating refractory diseases, including neuropathic pain. Other alternatives that can be used to inhibit corneal inflammation and pain include transient receptor of potential vanilloid-1 (TRPV-1) antagonists, such as resiniferatoxin, whose analgesic effects have been confirmed, and it also did not slow down the process of corneal wound healing in animal studies by blocking the sodium/calcium channels^(161)^.

#### Nanotechnology

There are several examples where delivery systems and microenvironment packing therapeutic molecules can render them more stable and available in the ocular tissue. Earlier, we mentioned the example of insulin topical therapy, although several other molecules are being designed and tested^(150,162)^. Another example is fungal keratitis (FK), a neglected disease (Orpha: 519930), which is strongly related to corneal trauma and has limited treatment options and poor prognosis^(163,164)^. FK therapy can also benefit from nanotechnology, where chitosan solutions or chitosan/poloxamer gel preparations for formulating the antifungal fluconazole, available for systemic use, can be an option for topical use, with corneal permeability and a sustained presence at the target sites^(165)^.

#### Cell reprogramming by gene therapy

Therapeutic strategies using cell reprogramming by gene therapy can promote the overexpression and local delivery of growth factors, anabolic hormones, or other intended adjuvant molecules to revert corneal inflammation or opacity, as detailed below. These therapeutic genes can reprogram the corneal cells or the LG^(166)^. Previous studies have shown that the salivary gland can be reprogrammed by viral vector gene therapy to work as a bioreactor and delivery system of hormones and other therapeutic molecules to treat severe oral dryness caused due to SS or radiotherapy at the experimental and clinical levels^(167-170)^. Furthermore, hormone gene therapy can be used to transfer the hormone erythropoietin (Epo), which preserved LG secretions and corneal epithelial integrity after the application of an adenovirus containing the Epo gene to the salivary gland^(139)^.

Corneal neovascularization reduces corneal transparency and the prognosis of corneal transplantation, and the actual treatment approaches for neovascularization are little effective and not long-lasting^(171,172)^. The key elements needed to prevent corneal neovascularization are a) constant vigilance for soluble vascular endothelial growth factor (VEGF) receptors on the ocular surface that can inhibit corneal neovascularization^(31)^ and b) the presence of corneal nerves working as anti-neovessel elements in the cornea^(83)^.

The neovascularization and opacity caused due to alkali burns in rat corneas were prevented in rats injected with an adenovirus containing the genes of soluble VEGF receptors (VEGFRs) in the LG. After 7 days, the corneas protected by the VEGFRs expressed in the LG were more transparent than those treated with an adenovirus with null genes or saline^(173)^. Therefore, LG may function as a target of gene therapy, functioning as a bioreactor for therapeutic molecules to prevent corneal scarring and blindness caused due to opacity or neovascularization^(173)^.

#### Tissue reengineering

Taking in account the limitations associated with OS reconstruction using the limbus transplant as mentioned above, the possibility of reengineering of corneal cells in vitro is being attempted. In the corneal limbus, the niches of stem cells exhibit mitotic activity mediated by at least three crucial transcription factors as follows: ATP-binding cassette, subfamily B, member 5 (ABCB5), paired box protein PAX6, and WNT7A^(174,175)^. Therefore, the strategies for preserving or restoring these niches could include cell reprogramming to overexpress these transcription factors to achieve a stable corneal epithelial layer to revert ulcers or keratinization and support the corneal epithelial layer. The approach of gene therapy using these transcription factors combined with tissue reengineering to grow distinct corneal layers *in vitro* opens the possibility of using the combined approaches to repair or replace corneal layers in therapies used for corneal wound healing^(176,177)^.

Biosynthesis and xenotransplantation of corneas have also been explored as possible alternatives to corneal transplantation using tissue reengineering^(114,178,179)^.

In cases where the LG is also damaged by the disease, the potential LG regeneration is limited^(180-182)^, and it is known that without the support of the LG, the corneal integrity is severely damaged^(183)^. Till date, only one study has been capable of demonstrating the restoration of a functional LG from transplanted embryonic tissue using tissue reengineering techniques^(184)^. Nevertheless, the strategies used for restoring and reintegrating extensively damaged LFU structures are unknown, which is probably the major challenge in reverting corneal blindness in the long-term in diseases involving the extraocular organs.

Table 2 summarizes the potential molecules and surgical interventions capable of working in a combined preventive and therapeutic manner or as an adjuvant therapy for corneal opacity to minimize the incidence of corneal blindness in the future (Table 2).

**Table 2:** Potential clinical and surgical novel strategies for corneal opacity treatment

Category		Treatment	Results	Author, year	
Combined Biological & Clinical Therapy		NK1R antagonists Lanepitant and Befetupitant for corneal neovascularization Contact lenses for the culture and delivery of corneal epithelial cells for the treatment of limbal stem cell deficiency	Reduction of corneal hemangiogenesis, lymphangiogenesis, and leukocyte infiltration Reconstruction of the recipient corneal surface	Bignami et al., 2014^(185)^ Brown et al., 2014^(186)^	
		Topical AMA0526 after corneal trauma	Inhibition of angiogenesis in vitro, reduction of corneal opacity, and neovascularization	Sijnave et al., 2015^(187)^	
		Topical applied cell-permeable FK506BP on corneal alkali burn injury	Corneal opacity and corneal neovascularization were significantly decreased	Kim et al., 2015^(188)^	
		Topical β-1,3-glucan in corneal alkali burn	Epithelial wound healing in vitro and suppression of acute inflammatory reaction	Choi et al., 2013^(189)^	
		Downregulation of vimentin by pharmacological agent withaferin A in corneal alkali injury	Vimentin deficiency alters the fibrotic response to corneal alkali injury and instead engages a reparative healing mechanism to restore corneal clarity	Bargagna-Mohan et al., 2012^(190)^	
		Inhibitory oligonucleotides of miR-206, miR-206-I, intrastromally injected into alkali-burned corneas. The possible binding of miR-206 on its molecular target Cx43 was assessed	Ameliorated inflammatory responses both in vivo and in vitro. Cx43 was directly targeted by miR-206	Li et al., 2015^(191)^	
		Injection of a naked plasmid expressing green fluorescent protein (GFP; pCMV-GFP) into an unwounded mouse corneal stroma. Injection of pCMV-GFP or plasmids expressing small hairpin RNA in the corneal wound injury model	In the corneal wound injury model, the GFP-positive cells demonstrated extensive dendritic-like processes that extended to adjacent cells, whereas the vimentin knockdown model showed significantly reduced corneal opacity	Das et al., 2014^(192)^	
		Application of angiogenin eye drops in neovascularization and corneal opacity	Reduction of the inflammatory response induced by TNF-α or LPS	Lee et al., 2016^(193)^	
		Keratocytes in culture and within intact normal and diseased tissue were induced to produce collagen type II upon treatment with TGFβ3 and dexamethasone	Collagen type II deposition and a threefold increase in corneal hardness and elasticity	Greene et al., 2016^(194)^	
		Fresh isolated omental cells were injected subconjunctivally in limbal corneal alkali injury	Reduction of corneal neovascularization and neutrophil infiltration	Bu et al., 2014^(195)^	
		Deep corneal neovessels treated with intrastromal injections of bevacizumab	Complete regression of neovessels in 16 patients, partial regression in 6 patients, and reduced opacity and improved visual acuity in 5 patients	Sarah et al., 2016^(196)^	
	Combined Biological & Surgical Therapy	Allogeneic limbal mesenchymal stem cell therapy after severe corneal chemical burn Autologous or allogenic cultivated limbal stem cell transplantation using a standardized protocol free from xenogenic products	Reduction of corneal opacity, neovascularization, and corneal fluorescein staining Reduction in corneal neovascularization	Acar et al., 2015^(197)^ Zakaria et al., 2014^(198)^	
		The transplantation of CECs in combination with the selective ROCK inhibitor Y-27632 in corneal endothelial dysfunction	Endothelium with a monolayer hexagonal cell shape with a normal expression of function-related markers; recovery of corneal transparency	Okumura et al., 2012^(199)^, Kinoshita et al.,^(158)^	
		Autologous and allogeneic limbal epithelial cells cultivated on amniotic membranes and transplanted in cases of limbal stem cell deficiency	Improvement in corneal epithelium quality, with subsequent improvement in symptoms, quality of life, and vision	Ramirez et al., 2015^(200)^	

Cx43= connexin43; TNF-α= tumor necrosis factor-alpha; LPS= lipopolysaccharide; NK1R= tachykinin 1 receptor; TGFβ3= transforming growth factor beta3; CECs= corneal endothelial cells.

Corneal blindness is a health problem and a therapeutic challenge. If few conditions have found efficient strategies as trachoma, which is being treated with the combined strategy that includes **S**urgery, **A**ntibiotics, **F**acial cleaning, and **E**nvironmental improvement (SAFE), and vitamin A supplementation can prevent keratomalacia secondary to nutritional problems even in remote regions, there are several conditions causing corneal blindness that are not being efficiently reverted by the currently available therapeutic strategies^(201,202)^. Novel therapeutic strategies using growth factors, anabolic agents, new promitotic, and anti-inflammatory drugs, combined with delivery systems, or corneal or LG genetic reprogramming of cells in association or not with corneal tissue reengineering can reduce the need for corneal transplantation and may function as adjuvants, providing customized therapies supporting more stable and long-lasting therapies for corneal transparency.
